# Preliminary experience with the EleVision IR system in detection of parathyroid glands autofluorescence and perfusion assessment with ICG

**DOI:** 10.3389/fendo.2022.1030007

**Published:** 2022-10-17

**Authors:** Petra Makovac, Mirza Muradbegovic, Timothy Mathieson, Marco S. Demarchi, Frédéric Triponez

**Affiliations:** Department of Thoracic and Endocrine Surgery, Geneva University Hospitals and University of Geneva, Geneva, Switzerland

**Keywords:** thyroid, thyroid surgery, near infrared fluorescence, endocrine surgery, fluorescence, autofluorescence

## Abstract

**Background:**

Postoperative hypoparathyroidism remains the most frequent complication of neck endocrine surgery. In order to reduce the incidence of this feared complication, several systems for imaging of near infrared autofluorescence (NIRAF) have been invented to help surgeons identify parathyroid glands (PTGs) and evaluate their vascularization. We evaluated the efficacy of the EleVision IR system in thyroid and parathyroid surgery.

**Methods:**

We used the EleVision IR system in 25 patients who underwent thyroid/parathyroid surgery or both at our institution between December 2020 and July 2021. At various stages of the surgery, the surgeon first looked for PTGs with the naked eye and then completed the visual inspection with NIRAF imaging. We then compared both the naked eye and NIRAF-supported PTGs detection rates. At the end of surgery, we performed indocyanine green angiography of PTGs in 17 patients.

**Results:**

In total, we identified 80% of PTGs: 65% with the naked eye only and additional 15% with the assistance of the EleVision IR system. 14 of 17 patients evaluated by ICG angiography had at least one well-vascularized PTG. Only one of these patients (a case of subtotal parathyroidectomy for tertiary hyperparathyroidism) developed symptomatic postoperative hypocalcemia despite a normal parathormone level. The three other patients had at least one remaining moderately-vascularized PTG and only one patient developed transient postoperative hypoparathyroidism.

**Conclusion:**

We concluded that EleVision IR provides an efficient support for identification and evaluation of PTGs, and may be of great assistance in endocrine surgery. The images are easy to interpret even for less experienced surgeons thanks to the different types of color visualization and the possibility to measure the relative fluorescence intensity of PTGs and surrounding tissues.

## Introduction

When performing thyroidectomy, it is crucial to avoid or minimize injury to the recurrent and superior laryngeal nerves as well as the parathyroid glands (PTGs). PTG damage, devascularization and/or inadvertent resection may lead to postoperative hypocalcemia, which is the most frequent complication after total thyroidectomy, occurring in 20-30% of patients and remaining permanent in 1-4% of patients (1). Permanent postoperative hypocalcemia caused by hypoparathyroidism may cause significant morbidity and even be life-threatening (2). Moreover, it may increase the length of hospital stay by imposing substitutive treatment thus impairing the quality of life.

The naked-eye identification and therefore preservation of PTGs is not always possible and often depends on a surgeon’s experience. Inadvertent resection of parathyroid tissue during thyroidectomy is common: the reported incidence ranges from 3.7 to 24.9% (3). Once PTGs are identified, the surgeon might adapt the surgical procedure to preserve their vascular pedicles by performing capsular dissection or leaving some thyroid tissue in place.

When performing parathyroidectomy, it is crucial to identify and remove all diseased PTGs. Failure to do so leads to persistent hyperparathyroidism, increased bone loss and repeated surgeries (4, 5).

In 2011, contrast-free near infrared autofluorescence (NIRAF) of PTGs was discovered at Vanderbilt University (6). Upon excitation by near-infrared (NIR) light at the wavelength of 785 nm, the parathyroid tissue emits light at around 820 nm. *In vivo*, PTGs have up to 20-fold stronger autofluorescent properties than the thyroid tissue (7). So, on NIR fluorescence images, PTGs appear as bright spots which are easily distinguished from the surrounding thyroid, muscle or fat tissue. NIRAF imaging detects both normal and diseased PTGs (8) although diseased PTGs (adenoma and hyperplasia) tend to have a weaker autofluorescence signal then normal PTGs (9, 10).

The autofluorescence of PTGs persists *ex vivo* for as long as 150 hours to 2 years. It also persists under extreme temperatures, formalin fixation or proteinase activity, so PTG autofluorescence is not an adequate means of determining tissue blood supply or viability (11). The use of contrast dyes such as indocyanine green (ICG) is therefore mandatory to assess the PTGs blood supply and help predict postoperative viability and function (12, 13).

Since this discovery of autofluorescence of PTGs, different detection systems have been developed to enable intraoperative PTG identification. The early systems based on wide-field NIR cameras and grayscale display have been successfully used not only for the visualization of NIRAF, but also to assess the glands’ vascularization by fluorescent angiography. The more recent PTG identification system uses a fiber optic probe to collect autofluorescence signal, which is then converted into binary audiovisual feedback (14). However, this system cannot currently be used to evaluate the perfusion of the PTGs.

The new EleVision IR platform (Medtronic, Dublin, Ireland), which includes the VS3 Iridium System, enables quantitative and qualitative NIR fluorescence imaging and visualization in color. It can be used to visualize PTGs during thyroid and parathyroid surgery and to inspect blood flow to the PTGs by ICG angiography.

In this study, we described our preliminary experience with PTGs identification and ICG angiography during endocrine surgery using the new EleVision IR system.

## Methods

We included 25 patients, median age 52 (interquartile range 25 – 77), 17 women and 8 men who underwent thyroid surgery, parathyroid surgery or both at our institution between December 2020 and July 2021. Patient demographics, the type of surgery and the indication for the surgery are described in **Table 1**. The patients were included prospectively, depending on the availability of the EleVision IR on the scheduled day of surgery. All patients signed an informed consent form prior to surgery. The study was approved by the Institutional Review Board of the University Hospitals of Geneva (2021-01990).

**Table 1:** Demographic and surgery characteristics of patients who underwent thyroid and/or parathyroid surgery.

	Thyroid surgery (n = 16)	Parathyroid surgery (n = 7)	Thyroid and parathyroid surgery (n = 2)
Mean age (years)	50	56	59
Sex			
Male	6	1	1
Female	10	6	1
Indication			
Benign	10	–	–
Papillary carcinoma	2	–	–
Papillary carcinoma N+	3	–	–
N+ laterocervical and central	1	–	–
Hyperparathyroidism 1	–	6	–
Hyperparathyroidism 3	–	1	–
Multinodular goitre + HPT 1	–	–	1
Thyroid adenoma + HPT 1	–	–	1
Type of surgery			
Total thyroidectomy	5	–	–
Total thyroidectomy + CND + LND	3	–	–
CND + LND	1	–	–
Lobectomy	6	–	–
Lobectomy + CND	1	–	–
Unilateral PTX	–	6	–
Bilateral PTX	–	1	–
Lobectomy + PTX	–	–	1
Total thyroidectomy + PTX	–	–	1

HPT, hyperparathyroidism; CND, central lymph nodes dissection; LND, lateral lymph nodes dissections; PTX, parathyroidectomy; n, number of patients.

### Surgical procedures

For thyroidectomy, we medialized the thyroid lobe after isthmectomy, section of the middle thyroid vein and dissection of the upper pole. We searched for the recurrent laryngeal nerve using a neuromonitoring probe (NIM 3.0, Medtronic, Dublin, Ireland). We then carefully searched for PTGs with the naked eye and the NIR imaging systems. We expected to find 2 PTGs per side, hence 2 PTG for a thyroid lobectomy, 4 PTGs for a total thyroidectomy. Once the PTGs and their precise anatomical features had been exposed, meticulous dissection of the thyroid lobe was performed in order to spare PTGs with their blood supply. For total thyroidectomy, the same procedure was applied to the second thyroid lobe. If all PTGs had not been identified *in situ*, the EleVision IR system was also used on the resected thyroid specimen to search for any incidentally removed PTGs. PTGs identified in this way were reimplanted into the sternocleidomastoid muscle.

For either unilateral or bilateral parathyroidectomy, we started with a small midline incision. For unilateral parathyroidectomy, we typically searched for a single diseased PTG localized before the surgery. Once the PTG had been identified by eye and before it was removed, we verified its fluorescence signal using the EleVision IR camera. In one case, the EleVision IR system enabled us to identify a second PTG localized in the illuminated surgical field. In the cases of parathyroid adenomas, the system was also used to verify the presence of the adenoma “cap” (10) on the excised specimen before it was sent to pathology. One patient included in this study underwent a bilateral cervical exploration with subtotal parathyroidectomy of four glands for parathyroid hyperplasia. In this case, before performing definitive parathyroidectomy, we evaluated the vascularization of the PTG remnant we planned to leave in place by ICG angiography.

### NIRAF imaging

As described above, during the surgery, at different steps of the dissection, the surgeon first looked for PTGs with the naked eye. The initial number of identified glands was recorded. Next, the EleVision IR system was used to confirm the correct identification of PTGs and to enable identification of PTGs that had not been previously detected. The total number of identified glands was noted.

**Figure 1:**
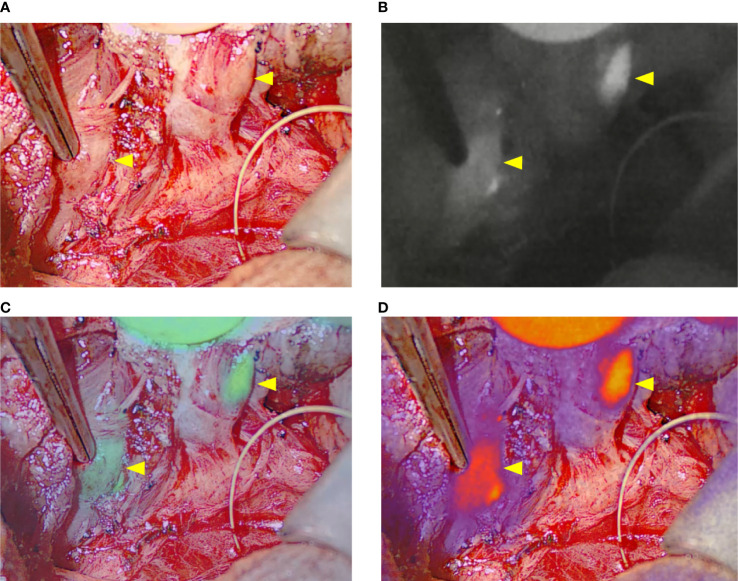
The four visualization modes of the EleVision IR system. **(A)** white light image; **(B)** black and white near infrared fluorescence image; **(C, D)** overlay of white light image and fluorescence image with a monochrome **(C)** or divergent **(D)** color map applied; yellow arrows indicate parathyroid glands.

The EleVision IR system contains a light source which delivers excitation light at 785 nm to the surgical field, a wide-field NIR camera that captures emitted light at 800 – 850 nm, and a screen. The system can measure relative fluorescence intensity and present it in percentages in real-time. The system has different visualization modes: white light image only, fluorescence image only or an overlay of white light and pseudocolor fluorescence images (**Figure 1**).

The NIR imaging camera was covered with a sterile drape and positioned above the patient or left on the surgical instrumentalist table to allow the surgeon to manipulate it during surgery. The NIR camera is sensitive to white light. To optimize the imaging, the operating room lights were turned off, sunlight blocked, and any other light sources (screens, head lamps) covered or turned off.

**Figure 2:**
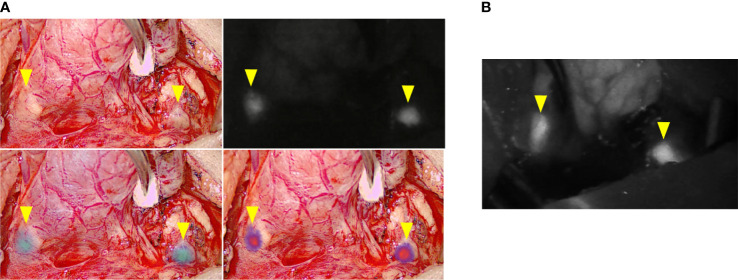
Comparison of intraoperative images generated with the different fluorescence imaging systems. **(A)** EleVision IR; The images are arranged as in **Figure 1** **(B)** FLUOBEAM LX; yellow arrows indicate parathyroid glands.

In five cases, we compared images generated with the EleVision IR and the FLUOBEAM LX systems (Fluoptics, Grenoble, France) (**Figure 2**).

### ICG angiography

**Figure 3:**
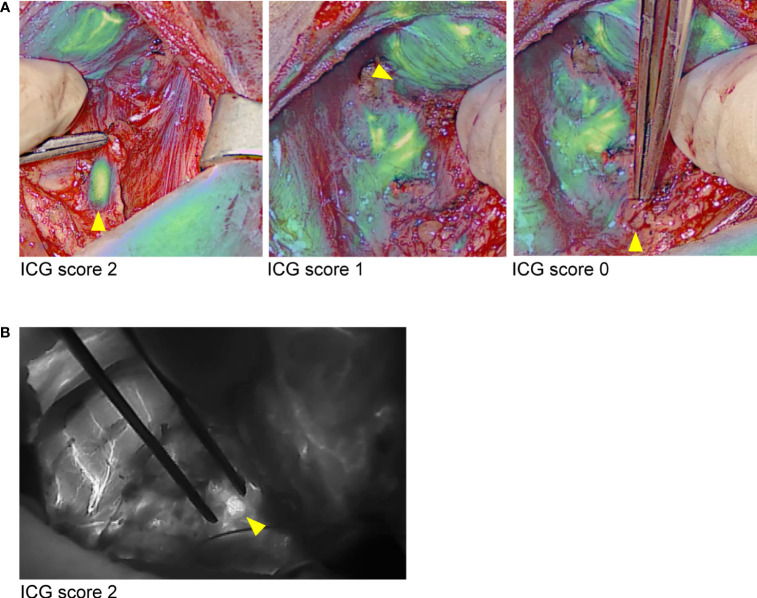
Comparison of images generated during ICG angiography with the different fluorescence imaging systems. **(A)** EleVision IR; Overlay of white light and monochrome fluorescence images; **(B)** FLUOBEAM LX; yellow arrows indicate parathyroid glands.

In 17 patients, at the end of the surgery, an angiography of PTGs was performed after intravenous injection of ICG as previously described (15). The EleVision IR system was then used to obtain angiography images and the parathyroid vascularization was scored from 0 to 2 (**Figure 3**; **Table 2**). An ICG score of 2 means a well vascularized gland, 1 means a moderately vascularized gland, and 0 means a devascularized gland (15, 16), as previously described. We previously demonstrated that at least one well-vascularized gland is sufficient to avoid postoperative hypoparathyroidism (15). No adverse events related to ICG injection or application of the EleVision IR system occurred in this study.

**Table 2:** ICG score and postoperative hypocalcemia status of 17 patients whose PTGs were evaluated by ICG angiography using EleVision IR after thyroid and parathyroid surgery.

Surgery	Patients with max ICG score of 2	Patients with max ICG score of 1	Patients diagnosed with hypocalcemia
**Thyroid**			
Total thyroidectomy	5	0	0
Total thyroidectomy + lymphadenectomy	2	1	1
Lymphadenectomy	1	0	0
Lobectomy	4	2	0
**Parathyroid**			
Bilateral PTX	1	0	1
**Thyroid and parathyroid**			
Total thyroidectomy + PTX	1	0	0
**Total**	**14**	**3**	**2**

PTX, parathyroidectomy.

### Follow-up

Parathormone and albumin-corrected calcium levels were measured at the hospital’s bioanalytical laboratory. Hypocalcemia was diagnosed for albumin-adjusted calcium concentration <2.00 mmol/L and hypoparathyroidism was diagnosed for parathormone concentration <1.1 pmol/L. Patients diagnosed with hypocalcemia were prescribed oral calcium and 25‐hydroxyvitamin D supplements.

## Results

Patients included in this study belonged to one of three groups based on the surgical procedure they underwent. Group 1 patients underwent thyroid surgery with or without lymphadenectomy) group 2 patients underwent parathyroidectomy (unilateral or bilateral), and group 3 patients underwent simultaneous parathyroid and thyroid surgery (**Table 1**).

In total we identified 80% of PTGs: 65% with the naked eye only and 15 percentage points more with the EleVision IR system (**Table 3**). In group 1, we were able to identify 28 out of 48 PTGs with the naked eye only. Nine more PTGs were identified using the EleVision IR system. In group 2, we were able to identify all ten pathological PTGs with the naked eye only and used EleVision IR only to validate these identifications. In one case of unilateral parathyroidectomy we were able to identify a normal PTG next to the pathological one thanks to the EleVision IR system. In group 3, four PTGs were identified with the naked eye only and subsequently confirmed with NIRAF imaging.

**Table 3:** Number of parathyroid glands identified with the naked eye only and the EleVision IR system during thyroid and parathyroid surgery.

Surgery	Patients	Identified PTGs	Naked eye	EleVision IR
**Thyroid **	**16**	**37/48**	**28**	**9**
Total thyroidectomy	5	15/20	9	6
Total thyroidectomy + CND + LND	3	10/12	9	1
CND + LND	1	1/2	1	0
Lobectomy	6	10/12	8	2
Lobectomy + CND	1	1/2	1	0
**Parathyroid**	**7**	**11/11**	**10**	**1**
Unilateral PTX	6	7/7	6	1
Bilateral PTX	1	4/4	4	0
**Thyroid and parathyroid**	**2**	**4/6**	**4**	**0**
Lobectomy + PTX	1	1/2	1	0
Total thyroidectomy + PTX	1	3/4	3	0
	**Total (%)**	**52/65 (80%)**	**42 (65%)**	**10 (15%)**

PTG, parathyroid gland; PTX, parathyroidectomy; CND, central neck dissection; LND, lateral neck dissection.

For the five cases in which we used both the EleVision IR system and the FLUOBEAM LX system, we found no difference in the rates of PTG detection. Regardless of which detection system or combination thereof was used, not all PTGs were found: 11 were missed in group 1 and two in group 3.

NIRAF imaging was helpful in identifying PTGs both *in situ* and in the surgical specimen. In group 1, eight additional PTGs were identified *in situ* and one additional PTG in the lymphadenectomy specimen. The latter PTG was then re-implanted into the sternocleidomastoid muscle.

**Figure 4:**
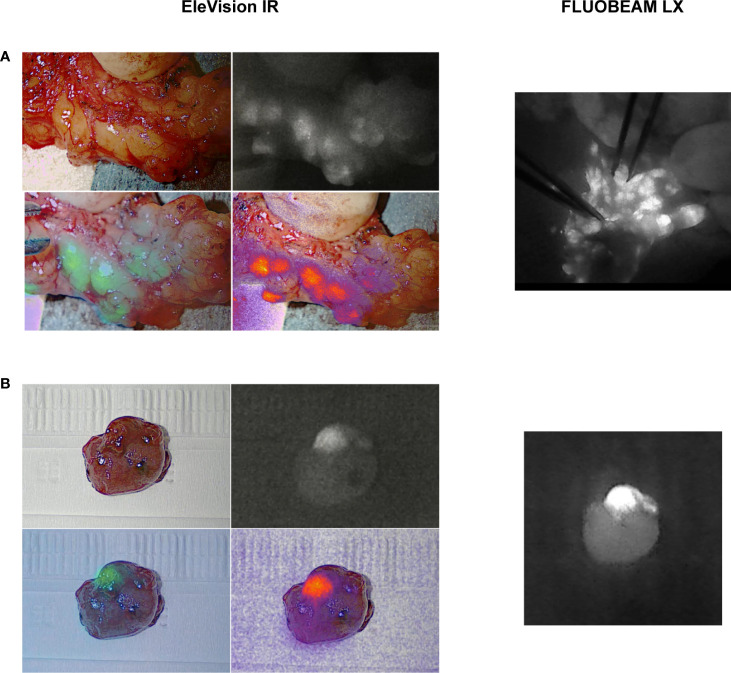
Comparison of images of ex vivo specimens generated with the EleVision IR and FLUOBEAM LX systems. **(A)** resected parathyroid adenoma; **(B)** pathological central lymph nodes in a lymphadenectomy specimen; The EleVision IR images in each panel are arranged as in **Figure 1**.

In the six cases of unilateral parathyroidectomy (group 2), we confirmed the presence of a single parathyroid adenoma by observing the clearly distinguishable adenoma cap with the EleVision IR system (**Figure 4B**). The cap corresponds to a rim of normal parathyroid tissue which emits stronger autofluorescence than the rest of the adenoma (10). In two patients, we compared the images generated with the EleVision IR and the FLUOBEAM LX systems (**Figure 4B**).

At the end of the surgery, ICG angiography of the PTGs using the EleVision IR system was performed in 17 patients. Of these, 14 had at least one PTG with an ICG score of 2 (good vascularization) (**Table 2**). Only one of these patients, the case of bilateral subtotal parathyroidectomy in which only half of a well perfused PTG was left in place, needed calcium supplementation at discharge for symptomatic postoperative hypocalcemia despite the normal parathormone level of 3.57 pmol/L. The three other patients had a maximal ICG score of 1 for at least one PTG left in place, and one patient needed calcium and vitamin D supplementation at discharge for a transient postoperative hypoparathyroidism (parathormone 1.0 pmol/L, albumin-corrected calcium 2.01 mmol/L), which lasted for 3 weeks in total. This patient had the 2 inferior PTGs reimplanted after CND and the 2 superior PTGs had an ICG score of 1. We also compared the ICG angiography images generated with EleVision IR and FLUOBEAM LX (**Figure 3**).

## Discussion

Identification of PTGs is a crucial step in endocrine surgery. Due to the high variability in anatomical location and vasculature of PTGs, their recognition is not always immediate. In thyroidectomy, when PTGs are not easily recognized, many surgeons prefer not to perform further dissection because of the risk of damaging delicate vasculature of PTGs. In such cases surgeons sometimes decide to perform an almost intracapsular thyroidectomy in the hope of saving the parathyroid vascular pedicles, which, nonetheless, does not always guarantee preservation of PTG function. In parathyroidectomy, omission of diseased PTGs in the first exploration may lead to repeated surgeries.

Using the EleVision IR system, we were able to identify 15 percentage points more PTGs than with the conventional visual examination only, despite the fact that all surgeries were performed by experienced endocrine surgeons using magnifying loupes, and gained greater confidence when dissecting the tissue. Combining direct observation with autofluorescence imaging, we identified 80% of PTGs. We also successfully used the system for ICG angiography assessment, which reliably predicts the absence of postoperative hypoparathyroidism in patients who have at least one well-perfused PTG (15). In the presented cases, only one patient developed transient postoperative hypoparathyroidism, which was predicted by the angiography results.

We found the EleVision IR system easy to use. The learning curve was shallow and the procedure added only few minutes to the surgery. However, care must be taken during imaging to maintain the correct distance (20 cm) and angle between the camera and the operating field to ensure optimal illumination and autofluorescence detection. In some cases, the imaging may be hindered by the length of the incision or a PTG lying too deep under opaque tissues. In such conditions, the surgeon is compelled to maneuver the device while watching the autofluorescence image on screen until the optimal image is obtained. Sometimes, especially with small incisions, it is not possible to capture both PTGs on one side of the neck in the same image. Unlike prior systems, however, the EleVision IR camera can be mounted onto an endoscopic device, which may improve maneuverability in cases of small incision, including for TOETVA.

In this preliminary study, we did not observed a quantitative difference in PTGs detection with EleVision IR and FLUOBEAM LX,

Falco et al. (11, 17,) were among the first to report that NIRAF imaging aids identification of PTGs during thyroid and parathyroid surgery. In their study, an average of 2.5 PTGs per patient were identified with the naked eye versus 3.7 per patient identified with NIRAF imaging. With the help of autofluorescence, all four glands were identified in 86% of patients versus in 12% with visual examination alone. Benmiloud et al. (18) demonstrated that the use of the autofluorescence-detecting devices was associated with an increased mean number of identified PTGs, a reduced rate of parathyroid autotransplantation, and a significantly lower rate of immediate postoperative hypocalcemia (5.2% *vs*. 20.9%). A meta-analysis of six eligible studies with a total of 2180 patients showed that NIRAF imaging reduces the risk of transient hypocalcemia and may lower the rate of permanent hypocalcemia (19). The prevalence of transient hypocalcemia was 8.11% (40/493) and 25.19% (425/1687) in the NIRAF and naked eye groups, respectively. The prevalence of permanent hypocalcemia was 0% (0/493) and 2.19% (37/1687) in the NIRAF and naked eye groups, respectively.

The PARAFLUO multicenter randomized clinical trial (20) conducted on 241 patients concluded that the use of NIRAF during total thyroidectomy lowered the rate of transient postoperative hypocalcemia from 22% to 9%, as well as decreased PTG autotransplantation and inadvertent resection rates from 16% to 4% and from 14% to 3%, respectively. It contrast to other studies, the PARAFLUO trials reported no change in the frequency of permanent hypocalcemia. Also Kim at al (21). showed that, although the use of NIRAF imaging lowers the frequency of inadvertent parathyroidectomy (from 14% to 6%), it does not affect the frequency of autotransplantation and transient or permanent hypocalcemia.

Even within the same patient, not all normal PTG’s fluoresce with the same intensity. Despite this heterogeneity, Hartl et al. (22) reported an *in vivo* sensitivity of 98.1%, meaning that a true PTG will rarely be missed with the NIR camera. Kim et al. (23) showed that up to 92.8% PTGs can be identified using NIRAF imaging in the early stages of surgery, i.e. before surgical dissection and exposure of PTGs. Ladurner et al. (24) reported a sensitivity of 90.2% for an endoscopic NIR imaging device: out of 41 imaged glands, four glands in one patients were not autofluorescent enough to be detected.

We also have to consider the risk of false positives caused by autofluorescence in the surrounding tissues. PTGs fluoresce more brightly than the thyroid tissue when excited by NIR light, but in some cases brown fat, colloid nodules and pathological lymph nodes may fluoresce at a level close to that of normal PTGs. Brown fat is the main source of falsely fluorescent tissue that visually resembles PTG due to its brownish-yellow color and its location in the areas surrounding the inferior PTG in the central neck. Colloid nodules may be confused with a subcapsular or intrathyroidal PTGs in the fluorescence imaging, but are usually distinguishable from one another with the naked eye after performing a small incision of the tissue. In this study, we did not confirm the nature of an autofluorescent tissue with pathological analysis or PTH measurement. This can be considered as a limitation. However, all surgeries were performed by experienced endocrine surgeons and therefore the risk to mistakenly identify a PTG is very low. In a study on ICG angiography, B Lang et al. made a biopsy of all PTGs and found parathyroid tissue in 324 biopsies of 340 PTGs (95.3%) (25).

In cases of papillary carcinoma with nodal metastases in the lymphadenectomy specimens, the pathological nodes can also be autofluorescent (**Figure 4A**). Cystic lesions seem to fluoresce with a higher intensity, and visual inspection may aid in distinguishing metastases from PTGs (26). The differentiation between a pathological lymph node and a PTG is not always easy with the naked eye nor when using NIRAF imaging. In case of doubt, we preferred to either perform a frozen section of a small fragment to confirm the nature of the tissue prior to reimplantation, or not to reimplant the gland.

The measurement of the emitted light intensity relative to the surrounding tissues provides a highly reliable and reproducible evaluation parameter. EleVision IR is currently the only available camera-based system that can express autofluorescence intensity as percentage relative to the surrounding tissue, making PTG identification easier and more certain. While our ICG angiography scoring system has proven effective in predicting the absence of postoperative hypoparathyroidism (15), it relies on a subjective evaluation of the fluorescence images by the surgeon, which may vary depending on experience. Further calibration of the ICG angiography with the EleVision IR, could lead to the development of evaluation methods based on numerical thresholds.

In the future, optimized standardized protocols for fluorescence signal acquisition should be developed (27) and adequate staff training ensured to minimize added operative time and address technical difficulties of using the system. Such measures may lead to a higher sensitivity and specificity, and help eliminate some of the causes of false positives (19).

The cost-effectiveness of this technology in terms of reduction in morbidity versus overall costs and added operating time remains to be evaluated but the current literature contains many studies with high-level evidence in favor of routine use of this technology.

## Conclusions

The EleVision IR system enables more accurate identification of PTGs during endocrine surgery than visual examination alone. This helps avoid futile tissue dissection in search of PTGs and consequent accidental devascularization leading to postoperative loss of function. Although surgeon’s experience remains paramount, especially given the risk of false positives, we believe that the EleVision IR system may be of great assistance. The color-enhanced display of parathyroid autofluorescence and its quantification relative to the surrounding tissues substantially eases the identification of PTGs.

## Data availability statement

The original contributions presented in the study are included in the article/supplementary material. Further inquiries can be directed to the corresponding author.

## Ethics statement

This study was reviewed and approved by Institutional Review Board of the University Hospitals of Geneva (2021-01990). Written informed consent for participation was not required for this study in accordance with the national legislation and the institutional requirements.

## Author contributions

All authors contributed to conception and design of the study. MD and PM contributed equally and are joint first authors of this article. PM, MM, MD organised the database and performed the statistical analysis. MD and PM wrote the first draft of the manuscript. MD and FT reviewed and completed the draft of the manuscript in its definitive form. All authors contributed to manuscript revision, read, and approved the submitted version
