# Erratum: Preliminary experience with the EleVision IR system in detection of parathyroid glands autofluorescence and perfusion assessment with ICG

**DOI:** 10.3389/fendo.2023.1175349

**Published:** 2023-03-24

**Authors:** Frontiers Production Office

**Affiliations:** Frontiers Media SA, Lausanne, Switzerland

**Keywords:** thyroid, thyroid surgery, near infrared fluorescence, endocrine surgery, fluorescence, autofluorescence

An omission to the funding section of the original article was made in error. The following sentence has been added: “Open access funding was provided by the University of Geneva”.

The original version of this article has been updated.

